# A near-peer regional surgical teaching programme designed by medical students, delivered by junior doctors

**DOI:** 10.1080/10872981.2019.1583969

**Published:** 2019-03-29

**Authors:** A. Musbahi, A. Sharpe, R. Straughan, S. Ong, A. Alhaddabi, A Reddy

**Affiliations:** South Tees NHS Trust, James Cook Hospital, Middlesbrough, UK

**Keywords:** Near-peer assisted learning, surgical education

## Abstract

**Background**: Near-peer teaching initiatives has been shown to be a highly successful method of improving student learning. There has been little data on surgical teaching initiatives of this kind and little data to show if this improves student confidence in surgical topics. This study was designed to show whether a regional surgical teaching programme, delivered by junior doctors, improves confidence levels of students prior to their final examinations. **Method**: Final year medical students were invited from four hospitals in the Northern deanery of England to participate in a voluntary surgical teaching day. Junior doctors were then recruited to present on various surgical topics based on their own knowledge and experience of finals examinations and working on the wards. A pre and post-course questionnaire was designed, validated and distributed to the students to assess their confidence on a five-point Likert scale of 1–5 (1- most confidence, 5- least confidence) levels in each of the 11 chosen topics. Other variables were also measured relating to the topics including visual material, enthusiasm, content relevance and communication. **Results**: 53 students completed the questionnaire (n = 53). There were 31 females and 22 males with a mean age of 24.7. A mean level of confidence of 2.7 pre-course and 1.6 post-course showed an increase in confidence by 68.8%. All eleven topics covered showed improvement in confidence. General Surgical Principles showed the lowest improvement in confidence from 2.683 to 1.917 (p = <0.001) compared to endocrine which showed the maximum increased in confidence from 3.650 to 1.694 (p = <0.0001). Orthopedics showed an increased in confidence from 3.010 to 1.62 (p = <0.0001). **Conclusion**: Near-peer education designed by medical students and delivered by junior doctors is an effective way for improving confidence levels and test results prior to finals examination and is also valuable for junior doctors.

## Background

The GMC clearly highlight that it is a doctor’s duty to develop the skills and attitudes of a competent teacher [1–3]. Traditionally, teaching has been delivered by clinical lecturers interested and trained in education [1]. However, with increasing clinical pressures for consultants to treat more patients in less time, educational and teaching opportunities are embraced by junior doctors [4]. Junior doctors are defined by us in this study as foundation year one and two who are one to two years post graduation but not sub-specialised, akin to interns in the USA.

Much research which has shaped modern medical teaching has involved Malcolm Knowles’ principles of ‘andragogy’ which has been defined as the science behind adult learning. The principles revolve around establishing an appropriate learning environment, involving learners in planning learning content as well as shifting the learning process to be more self-directed [5].

Near-peer teaching is a new method defined as teaching delivered by students or junior doctors who are one to two years ahead in their training [6]. It has proven to be a successful method of improving knowledge, confidence and clinical skills in domains such as prescribing, clinical examinations and communication skills [4,6,7]. Near-peer teachers are more familiar with the assessment process and the difficulties that students face and previous studies have reported great satisfaction amongst students [1,6]. Near-peer teaching is not only cost-effective but offers junior doctors chance to develop leadership and teaching skills as well as deliver students valuable support and knowledge [6].

Reflection is also an important process that all students and trainees are encouraged to do throughout their education and training [2,8,9]. It involves recognising and acknowledging situations that they have both coped well or struggled with and formulate action plans for continual development. It is important that educational opportunities are reflected on specifically so individuals can determine and further improve the efficacy of their work and learning [8]. In addition, students can provide feedback to their educators about how they can improve their teaching skills [9]. This study combines the importance of both teaching and this form of feedback.

The primary aim of this study is to determine the efficacy of a near-peer teaching programme in improving final year student confidence in broad surgical topics which was designed by final year medical students but delivered by junior doctors. Our hypothesis is that regional surgical teaching programmes delivered by Junior doctors benefit both the student and teacher and can improve test results.

## Method

### Organisation

The Surgical teaching programme was arranged over one day from 8:30 am to 5 pm at James Cook University Hospital Education Centre. Broad surgical topics were chosen based on final year curriculum and by consulting a small focus group of 10 fifth year students and voting on the final programme delivered six weeks prior to the programme delivery day. The topics chosen were general principles, upper & lower GI, hernias, bowel obstruction, vascular surgery, urology, orthopaedics, breast & endocrine and ophthalmology. Each topic was given a duration of 10 minutes with most of the 18 junior doctor teachers having prepared a slide presentation with time allocated for questions at the end but were given the autonomy to teach in any method they wished in the time given. Set times for breaks and testing were included in the programme.

### Recruitment

Fifty-three final year medical students (fifth year) were recruited to the regional teaching programme. These were final year medical students preparing to sit the final exams prior to graduation from medical school and entering the foundation programme. The teaching programme was advertised via emails to all final year medical students, of which there were 240 enrolled at the University, and also via word of mouth and posters in communal areas in the hospital as well as from University administration staff. Participation was voluntary. Only 50 places were available initially on the programme due to lecture theatre space, however on the day, 53 students turned up and so exceeded the cap by 3 students. Inclusion criteria included enrolment in final year studies at Newcastle University. There were no specific exclusion criteria and re-sit students were not excluded from the study.

### Teaching programme feedback

Feedback was collected in paper format anonymously from students and Junior Doctors by the project leaders. Feedback forms were given post-programme. These included sections on demographics, teacher confidence, student concerns, study sources, the rating of each teaching topic and the overall relevance of the course. The demographic section included questions on age, gender, previous degree, ethnicity, career aspiration and the consideration of a surgical career (if not considering a surgical career the reason for this).

Students were asked specific questions about their concerns and were asked to rate confidence levels with written papers, OSCE (manned and unmanned) and becoming a junior doctor after exams. They were also asked to rate their current sources of their revision, including course notes, textbooks, online websites and online multiple-choice questions.

Students rated each individual topic on the clarity of the learning objectives, content relevance, visual materials, teacher enthusiasm, communication, and student confidence after teaching. Regarding the overall relevance of the course they were asked to rate how useful the curriculum was, the relevance to the learning and the appropriateness of settings using 5-point Likert scale and free text comments.

The Likert scale was arranged from 1 to 5. A score of 5 indicated no confidence at all in this subject matter, a score of 4 indicated the student was slightly confident. A score of 3 was indeterminate or somewhat confident and a score of 2 was fairly confident. A score of 1 indicated complete confidence in the subject matter, Appendix 1.

Junior doctor teachers were given a separate feedback form to assess their motives for taking part and benefit acquired by participating. Information was also collected about time taken for preparation, resources used and prior teaching experience Appendix 2.

Results from the feedback surveys were collected and collated on an Excel Spreadsheet file and was analysed using Minitab Express (1.2.0, 2014, UK).

### Testing

Students were tested ten minutes prior to the commencement of the teaching programme and ten minutes after the end. The mini-test was created using a question bank of multiple-choice questions provided by each junior doctor teacher prior to the teaching programme. Each junior doctor teacher was asked to provide three questions on a surgical topic they were teaching. This created a bank of 33 questions. These were collated and a computer was used to randomly pick one question from each topic to make up a total of 11 questions for the mini-test. This was done to avoid bias towards one particular topic.

The junior doctor teachers were asked to provide the questions in MCQ or Multiple Choice Question format and provide 3 to 5 options for each question. This was in line with the University testing system and was also chosen as it would be easier to correct than a free text answer.

Eleven questions were randomly selected to conform to time limits to form the mini-test and also to cover all the topics taught (11 topics, 1 question per topic). These were distributed prior to the programme commencing to all students who completed it under timed test conditions (pre-test). A separate mini-test (post-test) using the same questions was completed at the end of the programme again under timed test conditions using the same questions as the pre-test to allow direct comparison. The tests were corrected by a programme director who was not involved in the teaching of the topics and who was a subspecialty surgical doctor. The results of both pre-programme testing and post-programme testing were compared and statistically analysed using Chi-squared and paired T-tests and where a p < 0.05 conferred statistical significance.

## Results

Fifty-three final year medical students six weeks from sitting final exams attended the Surgical teaching programme. The year group cohort had 240 students, however, 50 students were the target population due to resource limitation. Eighteen teachers were recruited voluntarily after advertising to junior doctors at the hospital to teach on the programme chosen topics by a focus group, see Table 1: Demographics.

**Table 1.** Demographic variables.

Student Demographics (*N* = 53)		
***Demographic variables***		
Average		24.74
Gender	Male	22
	Female	31
Provision Degree	Yes	13
	No	40
Interested in career in Surgery	Yes	26
	No	27

When tested on their confidence in individual topics prior to the teaching programme and testing, a mean level of confidence of 2.7 pre-course and 1.6 post-course showed an increase in confidence by 68.8%. All 11 topics covered showed improvement in confidence when rated prior to the teaching programme and test and after the teaching programme and test. General Surgical Principles showed the lowest improvement in confidence from 2.683 to 1.917 (p = <0.0001) compared to endocrine which showed the maximum increased in confidence from 3.650 to 1.694 (p = <0.0001). Orthopaedics showed an increase in confidence from 3.010 to 1.62 (p = <0.0001), see Table 2. When asked to give further comments, 45% of the students gave written positive feedback including comments such as ‘relevant for stage’, ‘concise’ and ‘appropriate’.

**Table 3.** Comparison of pre-programme and post-programme test scores! *calculated using paired t-test.

	Pre-programme(*N* = 53)	Post-programme(*N* = 53)	p-value*
Score (/11)			
4	2	-	
5	4	-	
6	7	1	
7	14	7	
8	8	7	
9	12	16	
10	4	13	
11	2	2	
Mean Score	7.58	8.938	<0.005

**Table 2.** Confidence levels of students, pre-teaching and post-teaching.

	Pre-programme(*N*= 53) (SD)	Post-programme(*N* = 53)	p-value*	Effect Size **
Topic				
General Principles	2.683 (±0.99)	1.917 (±0.82)	<0.0001	0.843
Upper GI	2.99 (±0.90)	1.729 (±0.71)	<0.0001	1.556
Lower GI	2.548 (±1.18)	1.612 (±0.61)	<0.0001	0.997
Hernias	2.567 (± 1.18)	1.735 (±0.64)	<0.0001	0.877
Gallstone Pathology	2.394 (± 1.31)	1.56 (± 0.54)	<0.0001	0.832
Urology	3.125 (±1.20)	1.902 (±0.78)	<0.0001	1.208
Vascular	2.865 (±1.12)	1.451 (±0.54)	<0.0001	1.612
Orthopedics	3.01 (±1.03)	1.62 (±0.75)	<0.0001	1.442
Breast	3.029 (±0.84)	1.9 (±0.68)	<0.0001	1.478
Endocrine	3.65 (±1.00)	1.694 (±0.68)	<0.0001	2.287
Ophthalmology	3.64 (±1.28)	2.22 (±1.02)	<0.0001	1.227

*calculated using paired t-test.

**calcuated using cohen’s D.

Post-programme test results showed a marked improvement from an average pre-programme score of 7.58 to a post-programme score of 8.938, giving an average 12% improvement in test. The number of students achieving 100% doubled. Thirteen students achieved 10/11 post-programme compared to four students pre-programme, see Table 3.

Following participation, junior doctors reported an improvement in their teaching skills, confidence and own knowledge. All junior doctors agreed they would take part again and 75% felt they would benefit from a course on teaching skills prior to delivering a similar session.

### Qualitative feedback

Free text comments focused on the conciseness of the presentations, the utility of the presentations to the curriculum. Some of the feedback focused on the fact that the teaching programme would have been better suited delivered earlier in the academic year. More detailed ophthalmology topics rather than just one ‘overview’ presentation and more detailed orthopaedic themed topics rather than one ‘overview’ presentation also would have been better received.

## Discussion

The main findings of our study were that i) students’ knowledge and confidence levels in taught topics improved as a result of attending near-peer surgical teaching; ii) junior doctors teaching skills, confidence and knowledge improved as a result of delivering near-peer surgical teaching, and iii) junior doctors are enthusiastic to deliver near-peer education and improve teaching skills.

Although near-peer education is relatively novel it has been recognised that is a valuable and effective method of teaching [10,11]. Similarly, this study demonstrated the value of near-peer teaching to medical students, prior their final examinations, and to junior doctors.

Many students highlighted the value and usefulness of the programme through positive written feedback. In addition, students made suggestions that the programme would have been of benefit earlier on in their medical education.

On average, students rated confidence levels as low [in certain topics] prior to attending the course. As students were asked to suggest the topics for teaching, this enabled the programme to focus on students’ areas of perceived weakness and also encouraged them to reflect on their own knowledge, thus making the learning ‘student centred’.

The junior doctors involved in delivering teaching were within the first three years of postgraduate training and therefore are familiar with the level of knowledge required by a medical student taking final examinations. This allowed junior doctors to deliver relevant teaching at the appropriate level. Positive engagement may also be a factor in maintaining teaching faculty retention.

As teaching skills are not routinely taught to medical students, opportunities to develop confidence and skills in teaching for junior doctors are particularly valuable. Junior doctors reported that their teaching skills, confidence and own knowledge improved as a result of participating in the programme. All tutors agreed they would participate in a similar programme again and 75% felt they would benefit from a course on teaching skills.

Another important aspect to consider in the delivery of the programme was the financial cost both to the students, teachers and organisers. Courses and revision materials can often be costly to the final year medical student and resources to deliver teaching programmes can also be of significant cost. The programme was held in a teaching hospital’s lecture theatre and junior doctors took part voluntarily, therefore the programme was provided at no cost to medical students, teachers or organisers.

Eighteen junior doctors delivered teaching at the programme, with 13 completing the post-programme feedback. All junior doctors volunteered to participate. As a relatively small cohort of junior doctors, it is unlikely to be an accurate representation of the whole population of junior doctors.

Medical students were invited to attend the surgical teaching programme, which was not a compulsory part of their course. Fifty-three students attended and it is likely that in choosing to attend the teaching programme that these students were more receptive to the teaching and may bias the results to show an overall greater benefit in the programme. Other reasons for non-attendance may be that the programme was held on one occasion only, it was held relatively near the examination dates and that students may prefer other methods of learning as this programme used a lecture-based approach.

### Future research

One of the aims of the programme was to improve student’s scores in final examinations. When delivering this programme in the future it may be of benefit to compare student’s final examination results to students who attended and did not attend the programme. Another improvement that may be of benefit to junior doctors would be to have a course or seminar on teaching methods prior to the programme as highlighted in the junior doctors’ feedback. Some students fed back that a similar course in an earlier stage of training would be useful, therefore holding the programme on multiple occasions could be of benefit and also improve attendance rates.

## Conclusion

A near-peer surgical teaching programme can deliver benefit to both students in terms of increased confidence and test results, as well as improvement to the teaching skills of the near-peer educator.

Competing Interests: The authors declare no competing interests.

## Appendix Appendix 1. Student Feedback Questionnaire

Designed by Medical Students, Taught by Junior Doctors: Feedback Questionnaire

By filling in this feedback form, you are providing us with consent to use this anonymised data for research purposes.

A. Demographics – *please fill in the blank space or circle Yes or No as appropriate (Y/N)*

(1) Age ____
(2) Gender – *please circle Male or Female*: M/F
(3) Previous degree not including intercalated degree: Y/N
(4) Ethnic background _____________________
(5) What are your career aspirations at the moment? _______________________
(6) Have you ever considered a career in surgery? Y/N
(7) If not, why not? _______________

B. Pre-course questions – *please rank the following*:

5 indicates no confidence at all in this subject matter

4 indicates you are slightly confident.

3 indeterminate or somewhat confident

2 fairly confident

1 indicates complete confidence in the subject matter

1. How confident do you feel about the following topics *(1 = most confident, 5 = least)*

General principles ____, Urology _____

Upper GI cancer _____ Orthopedics _____

Lower GI cancer and stomas _____ Breast surgery_____

Hernias and bowel obstruction _____ Endocrine surgery_____

Vascular surgery _____ Ophthalmology_____

Gallstones, diverticulitis., pancreatitis, appendicitis _____

2. Which do you feel most concerned about *(1 = most concerned, 5 = least)*
  - Mosler exam_____
  - Written exam_____
  - OSCE unmanned exam _____
  - OSCE manned exam_____
  - Becoming a F1 junior doctor_____
  3. Where do you get the most information from for revision *(insert number from 1–5, where 1 = source of most information, 5 = source of least information)*
    - Group learning *_____*
    - Course notes *_____*
    - Textbook *_____*
    - Online information *_____*
    - Online multiple choice questions *_____*

C. Post-course questions – *please rank from 1 −5 for each topic where 1 = excellent, 2 = good, 3 = average, 4 = below average, 5 = unsatisfied and please rank confidence as*:

5 indicates no confidence at all in this subject matter

4 indicates you are slightly confident.

3 indeterminate or somewhat confident

2 fairly confident

1 indicates complete confidence in the subject matter

***1 .*** General surgical principles
  - Clear learning objectives _____
  - Content relevance _____
  - Visual material *_____*
  - Enthusiasm of the teacher *_____*
  - Communication *_____*
  - Own confidence on the topic after this teaching _

***2 .*** Upper GI cancer Clear learning objectives *_____*

- Content relevance *_____*
- Visual material *_____*
- Enthusiasm of the teacher *_____*
- Communication *_____*
- Own confidence on the topic after this teaching _____

***3 .*** Lower GI cancer and stomas

- Clear learning objectives *_____*
- Content relevance *_____*
- Visual material *_____*
- Enthusiasm of the teacher *_____*
- Communication *_____*
- Own confidence on the topic after this teaching _____

***4.*** Hernias and bowel obstruction

- Clear learning objectives *_____*
- Content relevance *_____*
- Visual material *_____*
- Enthusiasm of the teacher *_____*
- Communication *_____*
- Own confidence on the topic after this teaching _____

***5.*** Gallstones, pancreatitis, appendicitis & diverticulitis

- Clear learning objectives *_____*
- Content relevance *_____*
- Visual material *_____*
- Enthusiasm of the teacher(s) *_____*
- Communication *_____*
- Own confidence on the topic after this teaching _____

***6.*** Vascular surgery

- Clear learning objectives *_____*
- Content relevance *_____*
- Visual material *_____*
- Enthusiasm of the teacher *_____*
- Communication *_____*
- Own confidence on the topic after this teaching _____

***7.*** Urology

- Clear learning objectives *_____*
- Content relevance *_____*
- Visual material *_____*
- Enthusiasm of the teacher *_____*
- Communication *_____*
- Own confidence on the topic after this teaching _____

***8.*** Orthopaedics

- Clear learning objectives *_____*
- Content relevance *_____*
- Visual material *_____*
- Enthusiasm of the teacher *_____*
- Communication *_____*
- Own confidence on the topic after this teaching _____

***9.*** Breast

- Clear learning objectives *_____*
- Content relevance *_____*
- Visual material *_____*
- Enthusiasm of the teacher *_____*
- Communication *_____*
- Own confidence on the topic after this teaching _____

***10.*** Endocrine

- Clear learning objectives *_____*
- Content relevance *_____*
- Visual material *_____*
- Enthusiasm of the teacher *_____*
- Communication *_____*
- Own confidence on the topic after this teaching _____

***11.*** Ophthalmology

- Clear learning objectives *_____*
- Content relevance *_____*
- Visual material *_____*
- Enthusiasm of the teacher *_____*
- Communication *____*_
- Own confidence on the topic after this teaching _____

D. Overall feedback

- Relevance to your learning
- Setting appropriate
- Would this teaching session be useful elsewhere in your curriculum Y/N

-If so where? *_________________________*

(iv) Further comments including suggested improvements?

## Appendix Appendix 2. Junior Doctor Teacher Feedback Form

**Form for teachers: Surgical Teaching Day**

1. Have you taken part in medical student teaching similar to the surgical teaching day before? (yes/no)
2. If so, how many sessions have you taught in before?
3. Do you think taking part in this surgical teaching day has improved your:

- teaching skills? (yes/no)
- knowledge? (yes/no)
- confidence in teaching? (yes/no)

4. Approximately how long did it take to prepare foryour teaching session?

- 1–2 hours
- 2–4 hours
- 4–6 hours
- >6 hours

5. What materials did you use to prepare for your teachingsession? (e.g., internet, course notes, textbooks etc)

6. What were your main reasons for taking part in theteaching day?

- To improve CV
- For interviews
- To improve teaching experience
- To revise own knowledge
- Other reason? Please specify below

7. Do you feel you would benefit from a course or seminaron teaching skills prior to delivering asimilar session?

- yes
- no

8. Would you take part in a teaching day similar to thisagain?

- yes
- no
